# Immunosuppressive effect of bladder cancer on function of dendritic cells involving of Jak2/STAT3 pathway

**DOI:** 10.18632/oncotarget.11434

**Published:** 2016-08-20

**Authors:** Weigang Xiu, Juan Ma, Ting Lei, Man Zhang, Shangyan Zhou

**Affiliations:** ^1^ Clinical Laboratory Medicine, Beijing Shijitan Hospital, Capital Medical University, Beijing, China; ^2^ Beijing Key Laboratory of Urinary Cellular Molecular Diagnostics, Beijing, China

**Keywords:** bladder cancer, dendritic cell, anti-tumor immunity, immunotherapy, Janus kinase 2/signal transducer and activator of transcription 3 pathway

## Abstract

Function of dendritic cells (DCs) is impaired by some cancer cells. However, the effect of bladder cancer cell (BCC) on phenotype and function of DCs remains unclear. In this study, healthy human peripheral blood mononuclear cells (PBMCs) derived DCs were co-cultured with BCC pumc-91 and adriamycin-resistant pumc-91/ADM. The expression of DC markers and costimulatory molecules decreased after co-culture. Co-cultured DCs rapidly underwent apoptosis, and had a declined capability to produce IL-8 and RANTES. Furthermore, co-cultured DCs showed impaired allogeneic T cell proliferation and T cell-derived cytokine secretion. Finally, AG490, a Jak2/STAT3 inhibitor, restored the expression of DC markers and costimulatory molecules. Of note, compared with control DCs, DCs co-cultured with pumc-91 produced more IP-10; DCs co-cultured with pumc-91/ADM secreted more MIG. Taken together, these results suggest BCC may inhibit maturation and function of DCs involving of Jak2/STAT3 pathway, and there may be different mechanisms by which adriamycin-resistant BCC restrains DC function in antitumor immune response.

## INTRODUCTION

Bladder cancer is one of the most common malignancies of urinary system, about 429800 new cases and 165100 deaths occurring in 2012 worldwide [1]. Bladder cancer can be categorized as non-muscle invasive bladder cancer (NMIBC) and muscle-invasive bladder cancer (MIBC). The characteristic of non-muscle invasive bladder cancer is frequent recurrence and low mortality. Moreover, 10–20% of non-muscle invasive bladder cancer will progress to muscle-invasive bladder cancer [2]. In the case of muscle-invasive bladder cancer, despite exist multiple treatment strategies, such as radical cystectomy, radiation therapy and chemotherapy, survival rates are poor [3]. These therapies restrict growth and development of tumor, however, they do not prevent recurrence and drug-resistant [4, 5]. Therefore, effective strategies are needed urgently.

Dendritic cells (DCs) are the most powerful antigen-presenting cells (APCs). To induce efficient lymphocyte response, antigen is bound to major histocompatibility complex (MHC) of DCs, then presented to and identified by T cells. In addition, DC vaccines highly express CD86, CD80, CD58 and CD40, and fully activate cytotoxic T-lymphocytes (CTLs) immune response against cancer cells [6]. Therefore, cancer immunotherapy involving of DCs acts as a promising method. The DC-based strategies not only prevent cancer recurrence, but also reduce its progression and metastasis [7–10]. Meanwhile, cytokines secreted from DCs play a vital role in antitumor response [11]. Moreover, tumor-infiltrating DCs (TIDC) are associated with predictable clinical outcome, prolonged survival and reduced tumor recurrence [12–14]. On the other hand, dysfunction of DCs can lead to ineffective T cell activation. In this case, T cell cannot recognize cancer cells and initiate defense response. Previous studies demonstrated that several malignant tumors could inhibit phenotype and function of DCs [15–17].

AG490 is a Jak2/STAT3 inhibitor, which can activate DCs by inducing cancer cell death [18], and suppress progression of bladder transitional cell carcinomas [19, 20]. Activated STAT3 signaling is related to bladder transitional cell carcinomas growth and survival [21]. Therefore, interrupting STAT3 signaling can suppress cancer drug resistance, growth and metastasis [22–24].

Until now, the effect of bladder cancer cell (BCC) on human DCs remains unclear. In our study, DCs were co-cultured with BCC pumc-91 and adriamycin-resistant pumc-91/ADM. The phenotype and function of DCs were evaluated thereafter. Moreover, we also investigated the immunosuppressive effect of BCC on DCs might involve of Jak2/STAT3 pathway.

## RESULTS

### Inhibition maturation of human DCs by co-culture with BCC

**Figure 1:**
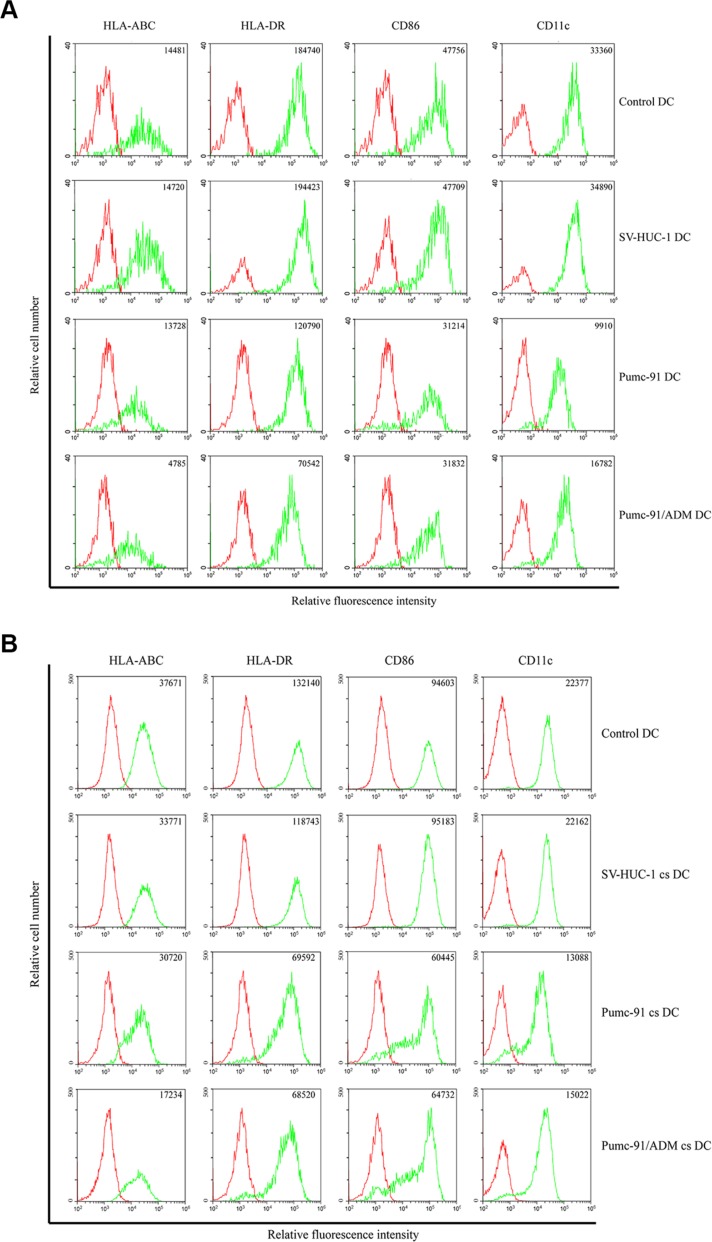
Effect of BCC on DCs phenotype (**A**) DCs were treated with SV-HUC-1 or BCC for 5 days and then induced with LPS for 24 h. The expressions of cell surface markers were analyzed by FACS. The mean fluorescence intensity (MFI) are present in the top right of each panel. Data shown are a representative experiment of three. (**B**) DCs cultured with supernatants of SV-HUC-1 or BCC. The cells were harvested and surface markers were analyzed by FACS. The mean fluorescence intensity (MFI) are present in the top right of each panel. Data shown are a representative experiment of three.

We first evaluated the effect of BCC on DC maturation. Compared with control DCs, pumc-91-exposed DCs displayed reduced expression of CD11c, HLA-DR and CD86. Besides that, the expression of HLA-ABC further decreased in DCs co-cultured with pumc-91/ADM. However, there were no differences between control DCs and DCs co-cultured with SV-HUC-1, a human uroepithelial cell line (Figure 1A). In supernatant experiment, compared with control DCs and SV-HUC-1 cs-exposed DC, the pumc-91cs-exposed DCs expressed lower levels of HLA-DR, CD11c and CD86, while the expression of HLA-ABC further decreased in pumc-91/ADM cs-exposed DCs. (Figure 1B). These data indicate that not only bladder cancer cell, but also the soluble factors from BCC inhibited the maturation of human DCs, and there might be different mechanisms by which adriamycin-resistant BCC inhibits DC maturation.

### BCC induced apoptosis of human DCs

**Figure 2:**
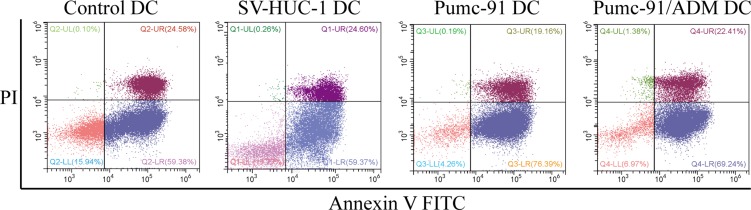
Increased apoptosis of DCs co-cultured with BCC Control DCs, SV-HUC-1 or BCC-exposed DCs were stained with FITC-Annexin-V and propidium iodide (PI). Apoptotic cells were increased in BCC-exposed DCs. Data shown are a representative experiment of three.

As cancer cells can escape immune surveillance by inducing DC apoptosis [25]. We next tested the effect of BCC on DC apoptosis. The apoptosis rate of DCs was assessed by the percentage of FITC-Annexin V positive cells, while the dead cells identified with propidium iodide. As shown in Figure 2, DCs co-cultured with BCC induced higher proportion of apoptotic cells in comparison to control DCs, and BCC promoted DC apoptosis.

### The effect of BCC on the production of DCs-derived chemokines

**Figure 3:**
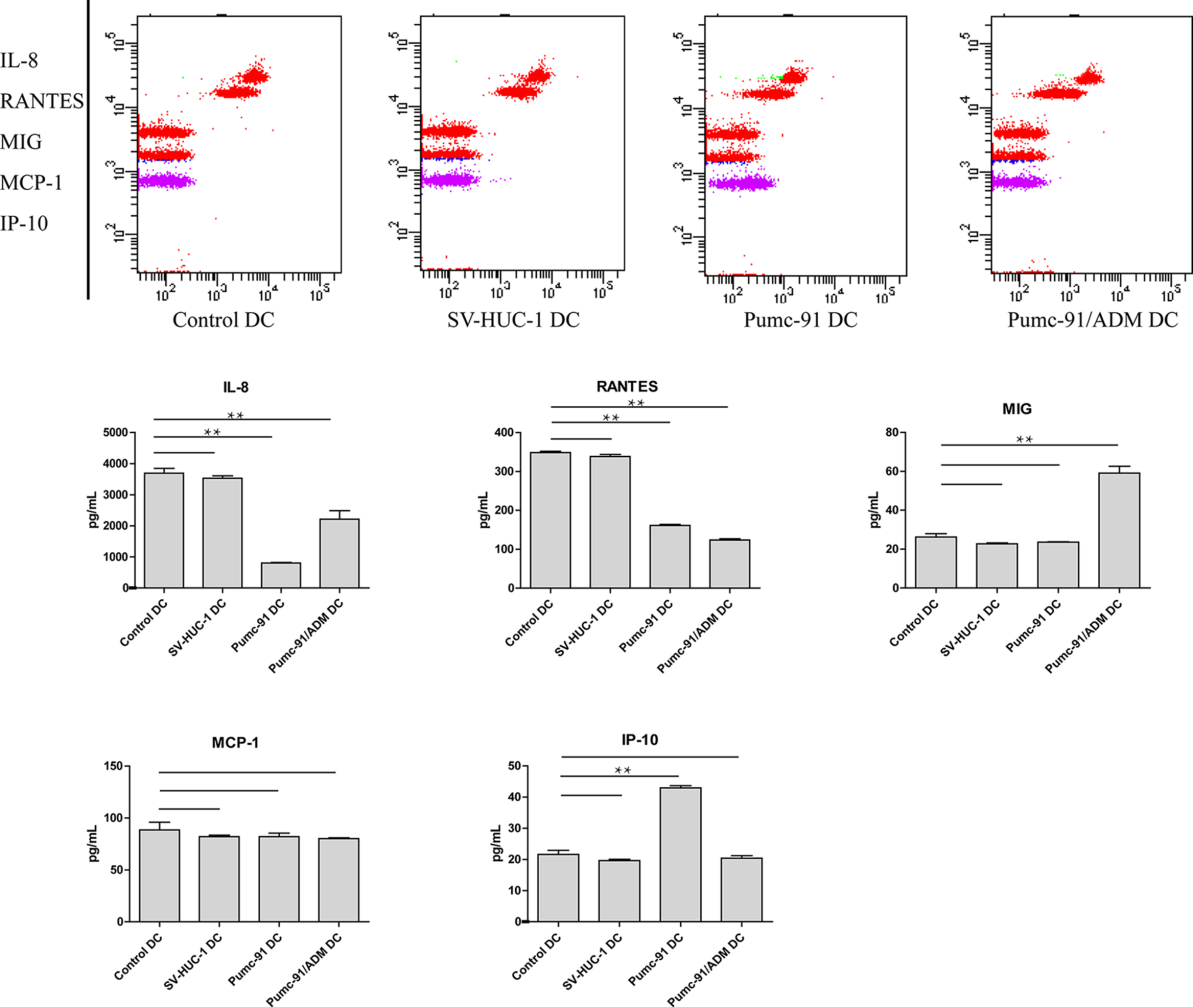
Production of chemokines IL-8, RANTES, MIG, MCP-1 and IP-10 by BCC-exposed DCs Control DCs, SV-HUC-1 or BCC-exposed DCs was induced by LPS. Cytometric Bead Array (CBA) was used to analyze concentrations of chemokines in cell-free supernatants. Values shown are mean ± standard deviation (SD) from triplicate representative experiments. **P < 0.01.

In addition to HLA molecules and co-stimulatory molecules, chemokines secreted from DCs also play an important role in antitumor immune response. Supernatants of control DCs, SV-HUC-1 and BCC-exposed DCs were analyzed for production of IP-10, MCP-1, MIG, RANTES and IL-8. Compared with control DCs and SV-HUC-1-exposed DCs, BCC-exposed DCs secreted lower levels of IL-8 and RANTES. Interestingly, compared with control DCs and SV-HUC-1-exposed DCs, DCs co-cultured with pumc-91 produced more IP-10, while DCs co-cultured with pumc-91/ADM secreted more MIG (Figure 3). These data demonstrate that BCC could affect the production of DCs-derived chemokines, and there might be different mechanisms by which adriamy cin-resistant BCC inhibits DC function.

### Inhibition of T cell proliferation by BCC-exposed DCs

**Figure 4:**
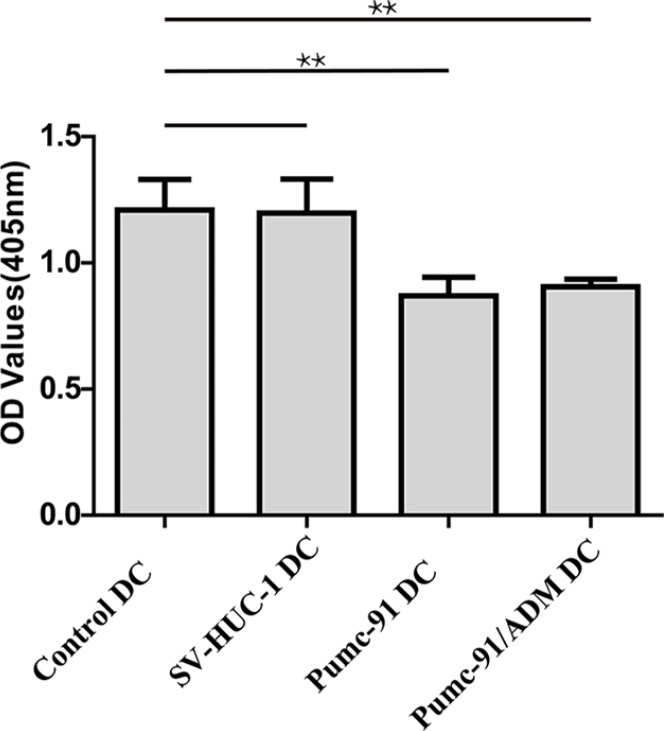
Reduced allogeneic T cells proliferation stimulated by DCs co-cultured with BCC Control DCs, SV-HUC-1 or BCC-exposed DCs were treated with LPS. Then purified and irradiated (30Gy) DCs were co-cultured with allogeneic T cells for 5 days. T cell proliferation was measured at 450nm by CCK-8. Values shown are mean ± standard deviation (SD) from triplicate representative experiments. **P < 0.01.

We next examined the immunostimulatory effect of DCs co-cultured with BCC. Control DCs, SV-HUC-1 and BCC-exposed DCs were used to stimulate allogeneic T cells. As shown in Figure 4, compared with control DCs and SV-HUC-1-exposed DCs, BCC co-cultured DCs showed impaired allogeneic T cell proliferation. These results demonstrated that BCC suppressed the capacity of DCs, leading to ineffective T cell activation.

### The effect of BCC-exposed DCs on T cell-derived cytokines

**Figure 5:**
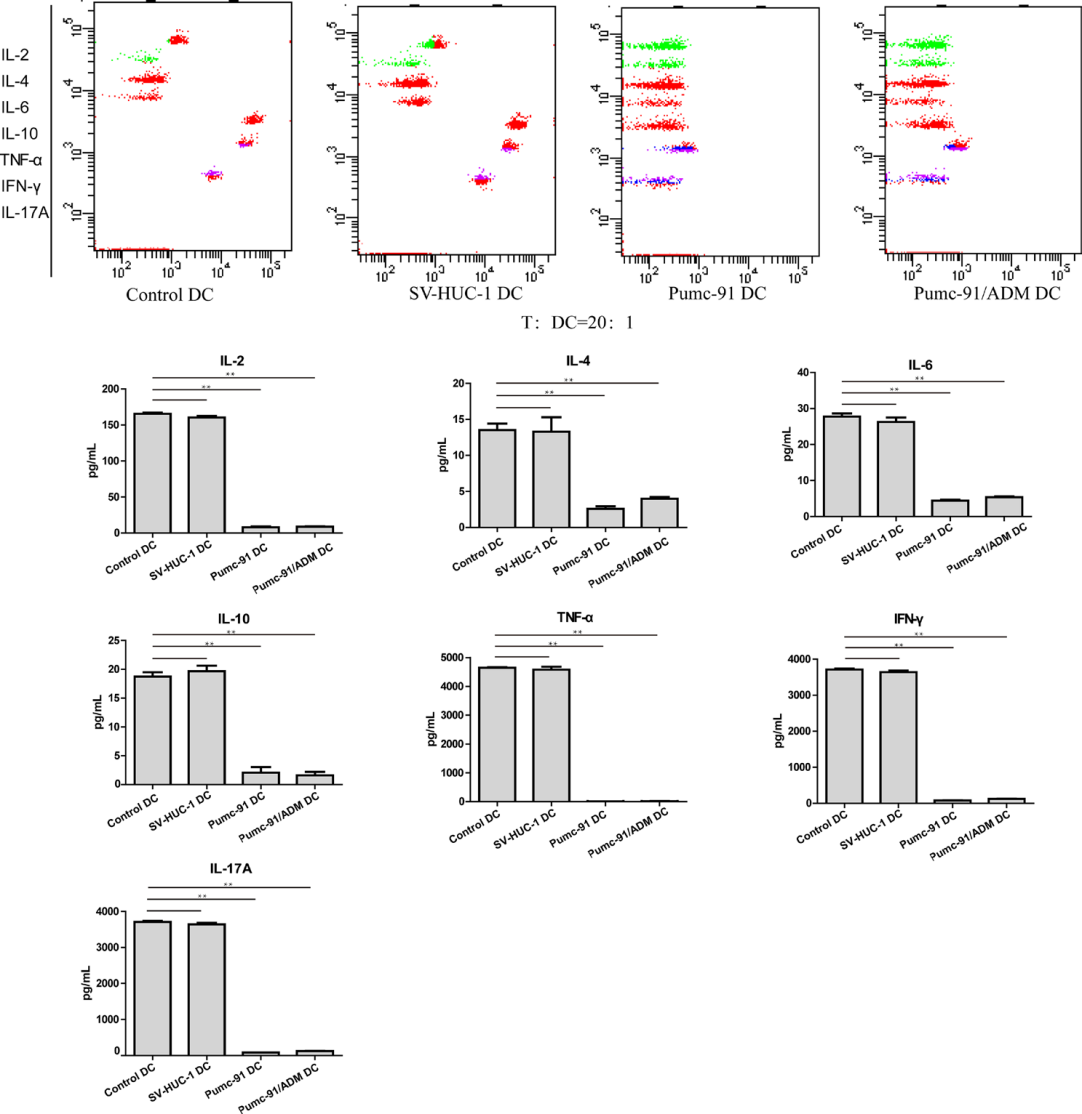
Reduced cytokine production of by allogeneic T cells induced by BCC-exposed DCs Control DCs, SV-HUC-1 or BCC-exposed DCs was treated with LPS. Purified and irradiated (30 Gy) DCs were co-cultured with allogeneic T cells for 5 days. The concentrations of cytokines in cell-free supernatants were measured by CBA human Th1/Th2/Th17 Cytokine Kit. Values shown are mean ± standard deviation (SD) from triplicate representative experiments. **P < 0.01.

To further examine the immunostimulatory effect of BCC-exposed DCs, we measured the levels of T cell-derived cytokines in above T cell-DC co-culture system by using the CBA human Th1/Th2/Th17 cytokine kit. We found that BCC exposed-DCs induced T cells secreting less cytokines than control DCs and SV-HUC-1-exposed DCs, including IL-2, IL-4, IL-6, IL-10, TNF-α, IFN-γ, and IL-17A (Figure 5). These data indicate that BCC-exposed DCs inhibited T cell to produce Th1, Th2 and Th17 cytokines.

### Immunosuppressive effect of BCC on DCs may through Jak2/STAT3 pathway

**Figure 6:**
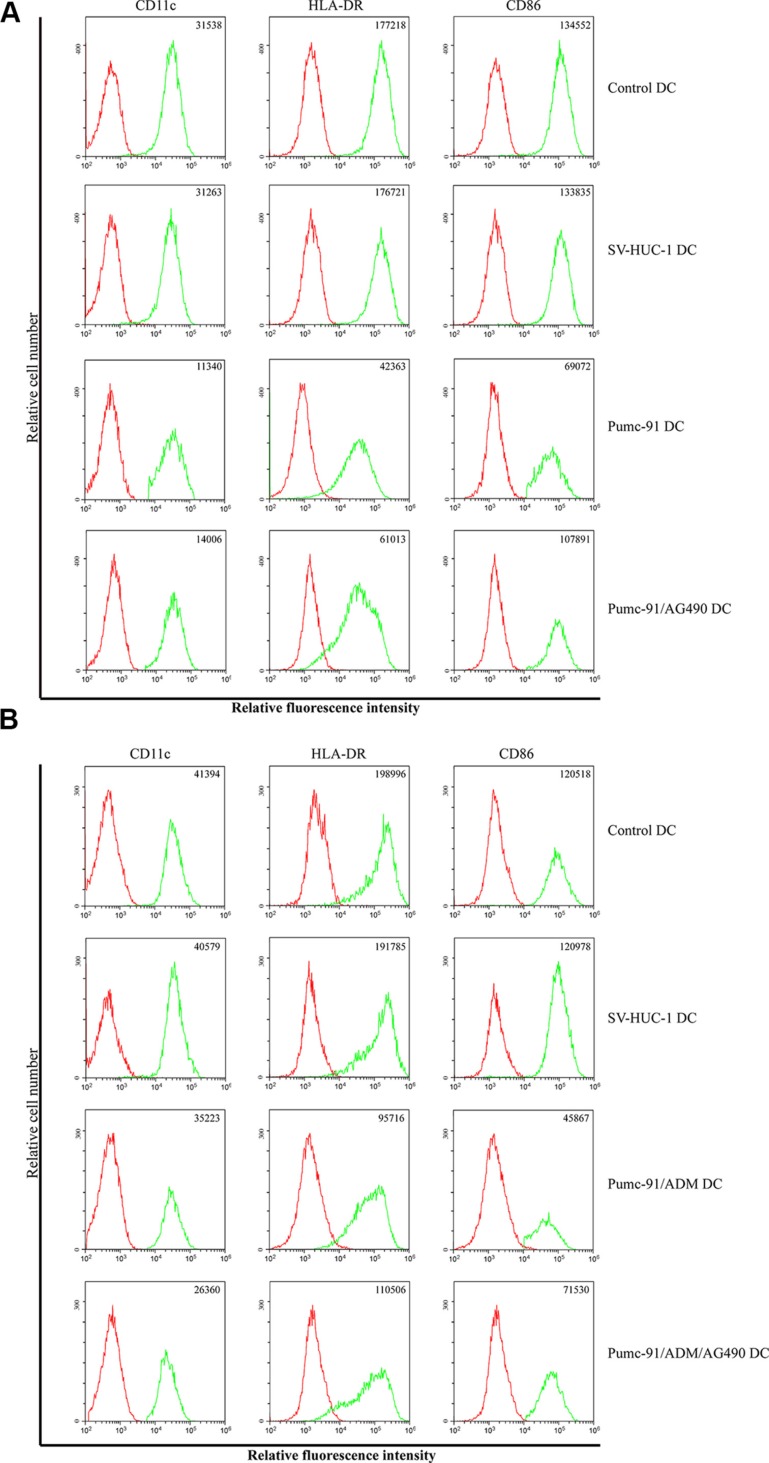
Effects of AG490 on BCC-exposed DCs phenotype Pumc-91 (**A**) or Pumc-91/ADM (**B**) prior treated with AG490 and co-cultured with DCs for 24 h, the expressions of DCs surface markers were analyzed by FACS. The mean fluorescence intensity (MFI) are present in the top right of each panel. Data shown are a representative experiment of three.

To explore the pathway of BCC inhibiting DC function. We prior treated pumc-91 or pumc-91/ADM with AG490, a Jak2/STAT3 inhibitor, and then co-cultured BCC with DCs. As shown in Figure 6A, compared with pumc-91-exposed DCs, the expression of CD11c, CD86 and HLA-DR were increased in DCs co-cultured with pumc-91/AG490; while in the pumc-91/ADM group, compared with pumc-91/ADM-exposed DCs, pumc-91/ADM/AG490-exposed DCs displayed increased expression of CD86 and HLA-DR (Figure 6B).

## DISCUSSION

Mature DCs activate tumor antigen-specific T cell to induce antitumor immune. However, immature DCs may lead to deficient activation of specific cytotoxic T cells, thus suppress antitumor immune response and promote cancer growth and progression [8, 10]. Recent studies reported that some cancers could induce immaturity and dysfunction of DCs. Uveal melanoma cell co-cultured DCs exhibited decreased expression of CD1a and CD83 and impaired T cell activation [16]. In our study, we identified the impairments in the phenotype of DCs co-cultured with BCC. We found that DCs exposed to BCC exhibited an immature phenotype. Compared with control DCs, DCs co-cultured with pumc-91 showed lower expression of CD11c, HLA-DR and CD86, while DCs co-cultured with pumc-91/ADM further displayed declined expression of HLA-ABC. In addition to the interactions between bladder cancer cells and DCs, DCs might be affected by soluble factors secreted from bladder cancer cells. DCs cultured with supernatants of pumc-91 and pumc-91/ADM showed similar impairment function. These data suggest that pumc-91 and pumc-91/ADM could suppress DC maturation. Interestingly, immunosuppressive effect of pumc-91 or pumc-91/ADM on maturation of DCs seems to be different. Of note, inhibition of DC maturation may be the characteristics of some tumors, while others not. Retinoblastoma cell could upregulate CD80 and CD86 on DCs, and stimulated allogeneic T cells proliferation [26].

A series of researches have been demonstrated that DCs undergo apoptosis after treated by cancer cells [25, 27]. Indeed, to evade antitumor immunity, cancer can induce DCs to undergo apoptosis, and thus lead to dysfunction of DCs [27]. BCC may utilize this mechanism to promote DCs apoptosis so that they could escape from immune surveillance. This may in part explain why T cells fail to become fully activated in our experiment.

As an effective inhibitor for Jak2/STAT3 pathway, AG490 reversed pancreatic cancer cell-induced inhibition of DC differentiation [28]. In our group, BCC decreased expression of costimulatory molecules on DCs, whereas BCC prior treated with AG490 could reverse the reduction of some molecules. This suggests that Jak2/STAT3 pathway, at least partly played a critical role in inducing immunosuppression effect of BCC. Furthermore, immunogenic apoptosis have a great effect on tumor microenvironment [29–31]. Compared to the ‘classical’ apoptosis, immunogenic apoptosis can induce DCs-based antitumor response. Bortezomib-killed tumor cells could be induced apoptosis, then activated DCs and enhanced antitumor T-cell response [32]. Prior treated with AG490 could cause apoptosis of lymphoma cells and promote DC maturation [18]. Accordingly, based on these findings, we speculate there may be same mechanisms by which AG490 effects on BCC and DCs.

CXCL8/IL-8 has multiple biological functions. On one hand, IL-8 led to aggressive growth and metastasis of human malignant melanoma [33]. On the other hand, IL-8 could inhibit growth and proliferation of some cancers [34, 35]. CCL5/RANTES is proinflammatory C-C chemokine. Several researches have demonstrated RANTES could delay or inhibit tumor growth and enhance the immunological antitumor effect [36, 37]. In our group, compared with control DCs, DCs co-cultured with BCC secreted less IL-8 and RANTES. Interestingly, compared with control DCs, pumc-91-exposed DCs secreted more IP-10, while pumc-91/ADM-exposed DCs produced more MIG. CXCL10/IP-10 is one of the Th1-attracting cytokines, and IFN-γ enhances expression of IP-10 during DCs maturation [38, 39]. IP-10 promoted invasion-related properties in human colorectal cancer cells [40]. Pumc-91 might utilize the properties to enhance its invasiveness. CXCL9/MIG induces tumor necrosis and exerts an important effect on antitumor response in Burkitt's lymphoma [41]. Conversely, increased MIG could facilitate melanoma cell metastasis through CXCR3 [42]. Accordingly, immunologic function of MIG may depend on the characteristics of cancer. MIG secreted from pumc-91/ADM-exposed DCs might trigger cancer migration and invasion. These findings implied that the immunosuppressive effect of pumc-91 or pumc-91/ADM on DCs might have different mechanisms. Moreover, our study also suggested that it is necessary to supply different immunotherapy methods for drug-resistant bladder cancer patients.

In our study, the expression of CD86 decreased in BCC-exposed DCs. Interaction of CD86 and CD80 with CD28 led to the activation and proliferation of T cells [43, 44]. Keep in line with these findings, we found that there was impaired allogeneic T cells proliferation when T cells were co-cultured with BCC-exposed DCs. Our study suggested that BCC could induce DC dysfunction, failing to induce T cell response. Moreover, BCC-exposed DCs underwent rapid apoptosis which may reduce the time window during which they interact with T cells [45]. In addition, we observed that T cells secreted less cytokines when co-cultured with BCC-exposed DCs. The data suggest that BCC restrain DCs' ability to activate T cells.

In summary, our study suggests BCC may inhibit maturation and function of DCs involving of Jak2/STAT3 pathway, and there may be different mechanisms by which adriamycin-resistant BCC restrains DC function in antitumor immune response.

## MATERIALS AND METHODS

### Cell culture

The SV-40 immortalized human uroepithelial cell line (SV-HUC-1) was obtained from Chinese Academy of Sciences Cell Bank (CASCB, China). SV-HUC-1 cells were cultured in Ham's F-12 (Sigma, USA) medium supplemented with 10% heat-inactivated fetal bovine serum (FBS) (Ausbian, Australia) at 37°C and 5% CO_2_. The human bladder cancer pumc-91 cell line was provided by Peking Union Medical College Hospital. The drug resistant cell line was pumc-91/ADM, which established by increasing the dosage of adriamycin gradually, and the final concentration of adriamycin was 1.0 μg/ml [46, 47]. SV-HUC-1 cell supernatant (SV-HUC-1cs), pumc-91 cell supernatant (Pumc-91cs) or pumc-91/ADM cell supernatant (Pumc-91/ADM cs) were prepared by seeding 10-cm dish (Corning, NY, USA) with 1 × 10^7^ SV-HUC-1 cell, pumc-91 cell or pumc-91/ADM cell in 10 ml of RPMI 1640 medium (Gibco, USA) for 24 hours and centrifuged to remove cells. Peripheral blood samples were from healthy individuals and peripheral blood mononuclear cells (PBMCs) were isolated immediate by Ficoll-Hypaque density gradient centrifugation. Subsequently, PBMCs were washed triple with AIM-V medium (Gibco, USA), suspended in AIM-V medium at 5 × 10^6^ cells/ml, and seeded in a 6-well plate (greiner bio-one, Germany) at 2 ml per well. The plate was incubated at 37°C for 2 h, and the non-adherent cells were discarded. The adherent cells were cultured for 6 days in AIM-V medium supplemented with 10% heat-inactivated FBS, 50 ng/ml rhGM-CSF and 50 ng/ml rhIL-4 (Peprotech, USA) to obtain immature DCs (iDCs). Half-volume medium exchange was performed every 3 days with AIM-V medium containing fresh cytokines.

### BCC treatment with AG490

AG490 (Sigma, USA) was dissolved in dimethylsulfoxide (DMSO) (Sigma, USA) and diluted to final concentrations of 200 μM. Pumc-91 or pumc-91/ADM was treated with AG490 for 24 h. The AG490-treated pumc-91 (pumc-91/AG490) or pumc-91/ADM (pumc-91/ADM/AG490) were washed twice and then co-cultured with DCs for 24 h. Background control group was 1.2‰ DMSO treated with pumc-91 or pumc-91/ADM.

### Co-cultured of dendritic cells with BCC

On day 5 of DCs culture, 1 × 10^6^ pumc-91 or pumc-91/ADM or SV-HUC-1 were added to each well of DCs. In a separate experiment, the cell-free supernatant of BCC was added to test DCs, while the same volume of AIM-V medium or SV-HUC-1cs was added to control DCs. On day 6, maturation of DCs was induced by 1 μg/ml of LPS (Sigma, USA) for 24 h. In most experiments, control DCs, BCC co-cultured DCs were washed and then purified with microbeads on QuadroMACS Starting kit (LD) using a Blood Dendritic Isolation kit (Miltenyi Biotech, Germany). The cell suspension was then passed through MiniMACS Starting kit (Miltenyi Biotech, Germany). According to the manufacturer's instruction, cells were labeled with the Non-DC Depletion Cocktail, comprising CD1c (BDCA-1), CD14 and CD19 microbeads for negative selected. Then cells were labeled with DC Enrichment Cocktail, comprising CD304 (BDCA-4/Neuropilin-1), CD141 (BDCA-3) and Biotin microbeads for positive selected. The DCs samples contain more than 95% CD1c^+^ DCs in these experiments.

### Flow cytometry analysis of DCs phenotype

Phenotype of DCs co-cultured with BCC was analyzed by FACS. The following test antibodies conjugated with either allophycocyanin (APC) or phycoerythrin (PE) was used: CD11c, CD86, HLA-ABC, HLA-DR and isotype control mAbs (eBioscience, San Diego, CA). The phenotype of DCs was tested by staining with antibodies for 30 minutes at 4°C, and then washed twice in phosphate buffered saline (PBS). Samples were analyzed by a flow cytometer (CytoFLEX, Beckman coulter) and data were processed using accompanying software (CytExpert, Beckman coulter).

### Apoptosis assay

To examine apoptosis, DCs were washed with ice-cold PBS, stained by FITC Annexin V Apoptosis Detection kit I (BD Pharmingen) according to the manufacturer's instruction. Samples were analyzed by the flow cytometer (CytoFLEX, Beckman coulter) and data were processed using the accompanying software (CytExpert, Beckman coulter).

### DC-derived chemokine assays

Purified DCs were cultured in 6 well plates at a density of 1.8 × 10^5^ cells/well in 2 ml of AIM-V medium. Cell free supernatant was harvested after induced maturation by using LPS. The level of IP-10, MCP-1, MIG, RANTES and IL-8 were measured by Cytometric Bead Array (CBA) Human Chemokine kit (BD bioscience) according to the manufacturer's instruction. Samples were analyzed using a flow cytometer (FACS Canto, BD Bioscience) and data were processed by accompanying software (FCAP Array; BD Bioscience).

### T cell proliferation assay

Heparinized peripheral blood samples were from healthy individuals and PBMCs were isolated immediate by Ficoll-Hypaque density gradient centrifugation. Pan T cell Isolation kit (Miltenyi Biotech) was used to separate CD3^+^ T cells according to the protocols recommended by manufacturer. X-irradiator (Gammacell 40 Exactor; MDS Nordion International, Inc., Ottawa, Ontario, CA) irradiated the purified DCs for 30 minutes at 30 G. Purified CD3^+^ T cells seeded into a round-bottom 96 well plate (greiner bio-one, Germany) at 2.0 × 10^5^ per well. Then purified CD3^+^ T cells were co-cultured with purified, stimulated and irradiated DCs at the ratio of 20:1. Background control group was T cells alone. The culture system was incubated for 5 days at 37°C in 5% CO2 in air. The proliferations of T cells were analyzed using Cell Counting Kit-8 (CCK-8) (Dojindo, Kumamoto, Japan) during the last 4 h of incubation.

### T cell-derived cytokine assays

Cell free supernatant was harvested from T cell-DC co-culture system after 72 h. The level of IL-2, IL-4, IL-6, IL-10, IL-17, TNF-α and IFN-γ were measured by Cytometric Bead Array (CBA) Human Th1/Th2/Th17 Cytokine kit (BD bioscience) according to the manufacturer recommendation. Samples were analyzed using a flow cytometer (FACS Canto, BD Bioscience) and data were processed by the accompanying software (FCAP Array; BD Bioscience).

### Statistical analysis

All the experiments were repeated three times. One-way ANOVA was used to estimate all analyses. *P* < 0.05 was accepted as statistical significance.
